# Screening instruments of cognition: The relation of the mini-mental state examination to the Edinburgh cognitive and behavioural ALS screen in amyotrophic lateral sclerosis

**DOI:** 10.1371/journal.pone.0304593

**Published:** 2024-06-20

**Authors:** Angela Serian, Julia Finsel, Albert C. Ludolph, Ingo Uttner, Dorothée Lulé

**Affiliations:** 1 Department of Clinical and Cognitive Neuroscience, Central Institute of Mental Health, University of Heidelberg, Mannheim, Germany; 2 Department of Neurology, Ulm University, Ulm, Germany; 3 German Center for Neurodegenerative Diseases (DZNE), Ulm Site, Ulm, Germany; New Jersey Institute of Technology, UNITED STATES

## Abstract

**Objective:**

The Edinburgh Cognitive and Behavioural ALS Screen (ECAS) is an established cognitive screening instrument for patients with amyotrophic lateral sclerosis (ALS). Different from tools like the Mini-Mental State Examination (MMSE), it is adjusted for motor impairment, yet, the latter remains one of the most widely used screening instruments, also in ALS studies. Thus, it is of utmost importance to relate outcome scores of both instruments to allow for comparison in ALS patients. This study reports on the performance of ALS patients in both tests with regard to incidence and degree of cognitive impairment, and the correspondence of both, ECAS and MMSE scores.

**Methods:**

We examined N = 84 ALS patients with the German versions of the ECAS and the MMSE. Performance in both tests regarding incidence and degree of cognitive impairment, and correspondence of frequency of cognitive impairment according to both tests was examined. The relationship between ECAS and MMSE scores was modelled with a non-linear regression model.

**Results:**

All ALS patients were able to complete the ECAS, 89.3% (N = 75) were capable to complete the MMSE. Prevalence of cognitive impairment was in both tests 22.7%, however agreement was only 52.9%. Despite, regression analyses yielded a strong positive relationship (adjusted R^2^ = .68) between the ECAS total score and the MMSE total score. Both tests were able to identify all patients with dementia.

**Conclusion:**

These results suggest that the MMSE is not ideal for cognitive screening in early-stage ALS patients. However, a rough translation of MMSE scores in ECAS scores is possible to estimate the cognitive performance level of patients, with the ECAS being more discriminative in the lower range of cognitive dysfunction (ECAS score: 80–136), for which the MMSE does not define cognitive impairment (corresponding MMSE score: 27–30).

## Introduction

It is now recognized that Amyotrophic Lateral Sclerosis (ALS) is a multisystemic disorder with neuropsychological dysfunction as the most common comorbidity in 35% to 45% of ALS patients [1]. These are being classified as cognitively impaired (ALSci; Amyotrophic Lateral Sclerosis with cognitive impairment) without fulfilling the criteria for dementia [1], while 5% up to 14% suffer from dementia, mostly in the sense of behavioural variant frontotemporal dementia (bvFTD) [1–3]. For ALSci, the most frequently reported cognitive impairments are deficits of the executive functions including verbal fluency and working memory as well as deficits of language and social cognition [4–6], depending on disease stage [7]. In bvFTD, behavioural symptoms such as personality change, irritability and pervasive deficits are the primary diagnostic criteria, although other cognitive impairments, especially in executive functions, may be present too [8]. These neuropsychological impairments may interfere with daily routine when they reach a certain threshold. Thus, for clinical practice it is essential to identify cognitive impairment and its severity.

To determine the cognitive status especially in ALS patients, the Edinburgh Cognitive and Behavioural ALS Screen (ECAS) was developed [9] and is now a well-established instrument with adaptations and translations for more than 20 languages [10]. It is designed to detect both ALS specific cognitive impairment, found in language, verbal fluency and executive functions, as well as ALS non-specific impairment in memory and visuospatial functions [9]. A major advantage of the ECAS is that it takes hand and speech motor impairment into account, allowing cognitive screening in severely physically impaired patients [9]. The most frequent form of dementia in ALS, bvFTD, can be determined with carer behaviour scale of the ECAS.

Although the ECAS was specially designed for ALS patients, in clinical practice and also in research with ALS patients, cognitive screening is often done via Mini-Mental State Examination (MMSE) which is one of the best known screening instruments validated for patients with neurological and psychiatric disorders [11, 12]. In our meta-analysis about cognition in ALS patients from 2022, we found that 31% of included studies used the MMSE for a cognitive screening or outcome measure, while only 20% of studies used the ECAS [6]. Reasons for the high application rate of the MMSE, even to screen ALS patients, may be its fast and easy application, its high acceptance among health professionals and researchers, its availability in multiple languages, and its use for the assessment of the severity of dementia [12–14]. However, the MMSE is motor dependent, so patients with progressive motor disability are unable to perform it in the course of their disease. Furthermore, the MMSE may be insensitive for clinical characteristics of frontotemporal dementia, like executive dysfunction or social cognition impairment in bvFTD [15], but some studies suggests that, nevertheless, it seems to be an appropriate measure to monitor severity and disease progression in bvFTD [16, 17].

The goal of this study was to compare the screening performance of the widely used MMSE and the specifically designed ECAS for cognitive dysfunction in ALS patients, to understand the relationship of both instruments, and to investigate the extent to which the test values of the ECAS correspond to the test values of the MMSE for cognitive impairment of ALS patients.

## Methods

### Participants

In total, 83 patients were recruited in the Department of Neurology at the University Hospital of Ulm between 21^st^ February 2018 and the 27^th^ May 2021. Interviews and screenings were performed by (neuro-)psychologists, health professionals and clinical neurologists in training. Total interview and screening lasted 30–60 minutes, depending on the physical impairments of the subjects. The study was approved by the Ethics Committees of the Ulm University (19/12), in accordance with the ethical standards of the revised Helsinki Declaration in 1983. All participants gave written informed consent prior to inclusion in the study. Inclusion criteria for the study were German as a first or comparably good second language, and no history of any psychiatric or neurological disorders, apart from ALS and (reactive) depression. We had to exclude one patient due to foreign first language and one patient due to rejected ALS-diagnosis. None of the patients had a clinical diagnosis of dementia at time of study inclusion, however two patients were afterwards diagnosed with dementia, one with FTD and one with primary progressive aphasia (PPA). In order to improve representation of patients with severe cognitive impairments up to dementia in the study, another three patients with ALS and suspected dementia were examined from 7^th^ March 2024 to 20^th^ March 2024. One of these patients received a clinical diagnosis of behavioural variant of FTD (bvFTD), for the other two patients, final findings regarding dementia development were still pending. In summary, 84 patients with the diagnosis of ALS according to the revised El Escorial criteria [18] could be included.

Demographics concluded of age, education, sex, months since symptom onset, site of onset, family history of ALS, severity of physical impairment (measured by the revised ALS Functional Rating Scale (ALSFRS-R, with a range from 48 (no impairment) to 0 (extreme impairment)), progression rate (calculated as 48 minus current ALSFRS-R score divided by months since symptom onset), usage of non-invasive ventilation (NIV) and/or percutaneous endoscopic gastrostomy (PEG), and depressiveness (measured by ALS-Depression-Inventory (ADI-12, with a range from 12 (no depression) to 48 (severe depression)) [19].

### Edinburgh Cognitive and Behavioural ALS Screen (ECAS)

The ECAS is a 136-point test consisting of 16 tasks covering the cognitive domains of language, verbal fluency and executive functions, subsumed under the ALS specific score, as well as memory, subsumed und the ALS non-specific score [9]. In this study, the validated German version of the ECAS was used [3] with established age- and education-adjusted cut-offs for all subscores and the total ECAS score to define cognitive impairment [20]. The ECAS can be performed either in a written or spoken version to minimize the influence of motor impairment. In this study, all participants performed the spoken version.

### Mini-Mental State Examination (MMSE)

The Mini Mental State Examination (MMSE) is a 30-point test consisting of 19 individual tasks covering orientation, calculation, memory, visuoconstruction and basic language and action tasks [11]. In this study the German version of MMSE was used [21]. Based on Jia et al. [22], the tasks were assigned to five domains, the tasks were subsumed under cognitive domains, namely orientation (orientation to place and time, 10 points), memory (repeat and recall 3 words, 6 points), visuospatial skills (copy intersecting pentagons, 1 point), language (naming, repetition, write a sentence, 4 points) and executive functions (serial 7 substraction, 3-step command test, reading command test, 9 points). Impairment of a cognitive domain was defined as -1 standard deviation of the mean. According to Kilada et al. [23], an overall MMSE score of less than 27 is considered a validated diagnostic standard for cognitive impairment. If patients were not able to complete a task of the MMSE due to physical decline and motor restrictions, they were grouped into a separate group “patients without motor capacity for MMSE” and their results of the MMSE were not used in the final analysis.

### Statistics

Data distribution of metric demographic and cognitive variables were tested with the Shapiro-Wilk test. Group differences between patients with motor capacity to complete the MMSE and patients without the motor capacity to complete the MMSE were tested with Chi-Square test for nominal data, with Mann-Whitney-U test for non-normal distributed data, and with two-tailed t-test for normal-distributed data. A threshold of α = 0.05 (two-tailed) was applied for statistical interference. Cohen’s *d* was used as effect size parameter in significant statistical tests with 0.2, 0.5 and 0.8 as thresholds for a small, medium, and large effect, respectively. Curve estimation regression (invers model, s-curve model, and cubic model) was performed to describe the relationship between MMSE and ECAS, with ECAS as predictor, choosing the best curve fit by the highest *R*^2^. The data was analyzed using R 3.6.3 and IBM SPSS statistics version 25.

## Results

### Demographics

Mean values and standard deviations (*SD*) of demographics of included patients are shown in Table 1. Thirty-six patients (42.9%) in this sample showed depressive symptoms according to a cut-off score ≥ 23 for a valid hint for depressive symptoms [24], while twelve patients (14.3%) showed clinical relevant depressive symptoms with a cut-off score >29 [24] assessed by ADI-12.

**Table 1:** Demographic data of ALS patients.

	All patients (N = 84)		Patients with motor capacity for MMSE (N = 75)		Patients without motor capacity for MMSE (N = 9)	
	M (SD) or N	Range	M (SD) or N	Range	M (SD) or N	Range
**Age (years)**	65.39 (10.39)	36–87	65.16 (10.61)	36–87	67.33 (8.57)	52–79
**Education (years)**	12.96 (3.74)	8–25.5	13.10 (3.92)	8–25.5	11.78 (1.18)	10–13.5
**Sex (m:f)**	53:31		49:26		4:5	
**Months since onset**	18.64 (19.48)	2–120	17.89 (20.00)	2–120	26.43 (11.01)	14–48
**Site of onset (bulbar:spinal)**	30:54		26:49		4:5	
**Family history of ALS (yes:no)**	6:78		4:71		2:7	
**ALSFRS-R**	38.34 (6.08)	23–48	38.83 (6.07)	23–48	34.33 (4.72)	27–42
**Progression**	0.86 (0.80)	0–4.67	0.89 (0.83)	0–4.67	0.56 (0.23)	0.23–0.79
**NIV (yes:no)**	15:69		13:62		2:7	
**PEG (yes:no)**	2:82		1:74		1:8	
**ADI-12**	22.87 (6.48)	13–40	22.77 (6.70)	13–40	23.67 (4.39)	18–33
**ECAS**	97.81 (21.21)	28–128	98.53 (20.43)	28–128	91.78 (27.57)	46–120

ALSFRS-R = revised ALS Functional Rating Scale. NIV = non-invasive ventilation, PEG = percutaneous endoscopic gastrostomy, ECAS = Edinburgh Cognitive and Behavioural ALS Screen, MMSE = Mini Mental Status Examination, ADI-12 = ALS-Depression-Inventory, M = mean, SD = standard deviation.

Nine participants (10.7%) were not able to complete the MMSE due to loss of needed motor capacity, their demographics are shown separately in Table 1. These patients had a longer disease duration (months of onset: U = 92.50, *p* = .006, *d* = 0.84) and a lower ALSFRS-R (U = 491.00, *p* = .03, *d* = 0.50), but did not differ in terms of age (*t*(82) = -0.70, *p* = .50), sex (χ^2^(1) = 0.74, *p* = .39), education (U = 386.00, p = .49), progression rate (U = 285.00, *p* = .62), family history of ALS (χ^2^(1) = 1.38, *p* = .24), NIV and PEG use (χ^2^(1) = 0.00, *p* > .99 and χ^2^(1) = 0.44, *p* = .51, respectively), depressiveness (U = 276.50, *p* = .38) and ECAS total score (U = 369.50, *p* = .65).

### Performance on ECAS and MMSE

The mean ECAS total score was 98.53 (*SD* = 20.43, range 28–128), 17 participants (22.7%) were under the respective German age- und education-adjusted cut-offs [3] for impaired cognition (Table 2). The mean MMSE total score in this sample was 27.68 (*SD* = 2.57, range 17–30). Seventeen participants (22.7%) showed a MMSE total score under the cut-off score of < 27 for cognitive impairment [23] (Table 2). A total of 9/75 (12.0%) participants showed impairment in both tests, while another 8/75 (10.7%) who tested normal in the MMSE were impaired in the ECAS, and 58/75 (77.3%) tested normal in both MMSE and ECAS (Table 3).

**Table 2:** Neuropsychological screening of ALS patients with motor capacity for ECAS and MMSE (N = 75).

Domain names	ECAS				MMSE			
	Item / Score	M (SD)	Range (possible Range)	Impaired^a^	Item / Score	M (SD)	Range (possible Range)	Impaired^b^
**Memory**	ECAS Memory Score	13.37 (4.99)	0–22 (0–24)	22.7%	Repeat 3 words, Recall 3 words	5.03 (1.02)	3–6 (0–6)	27.8% (cut-off ≤4.01)
**Visuospatial skills**	ECAS Visuospatial Score	11.32 (1.30)	6–12 (0–12)	18.6%	Copy intersecting pentagons	0.89 (0.32)	0–1 (0–1)	11.1% (cut-off ≤0.57)
**Language**	ECAS Language Score	24.61 (3.65)	12–28 (0–28)	18.6%	Naming (pencil, watch), repetition, write a sentence	3.83 (0.44)	2–4 (0–4)	13.9% (cut-off ≤3.39)
**Executive functions**	ECAS executive functioning	33.87 (8.62)	7–48 (0–48)	17.3%	3-step command test, reading command test, serial 7 substraction	8.46 (1.05)	4–9 (0–9)	11.1% (cut-off ≤7.41)
**Verbal fluency**	ECAS verbal fluency score	15.45 (6.31)	0–24 (0–24)	36.0%				
**Orientation**					Orientation to place and time	9.47 (1.05)	5–10 (0–10)	12.5% (cut-off ≤8.42)
**Total Score**	ECAS total score	98.53 (20.43)	28–128 (0–136)	22.7%	MMSE total score	27.68 (2.57)	17–30 (0–30)	22.7% (cut-off ≤26)

ECAS = Edinburgh Cognitive and Behavioural ALS Screen. MMSE = Mini-Mental State Examination. M = mean, SD = standard deviation.

^a^Impairment in ECAS domains and total score according to German age- and education-adjusted cut-offs (Loose et al., 2016.).

^b^Impairment in MMSE domains defined as -1 SD, impairment in MMSE total score according to Kilada et al., 2005.

**Table 3:** Agreement of ECAS and MMSE regarding cognitive impairment.

	Impaired in ECAS (age- & education-adjusted cut-off) in N (%)	Not impaired in ECAS in N (%)	Total in N (%)
**Impaired in MMSE (<27 points) in N (%)**	9 (12.0%)	8 (10.7%)	17 (22.7%)
**Not impaired in MMSE in N (%)**	8 (10.7%)	50 (66.7%)	58 (77.3%)
**Total in N (%)**	17 (22.7%)	58 (77.3%)	75 (100%)

MMSE = Mini Mental Status Examination. ECAS = Edinburgh Cognitive and Behavioural ALS Screen.

As seen in Table 2, between 17.3% (executive functions) to 36.0% (verbal fluency) of patients were impaired in the individual domains of the ECAS, while between 11.1% (visuospatial skills and executive functions) and 27.8% (memory) were impaired in individual domains of the MMSE. Dysfunction in verbal fluency, which was only measured by the ECAS, was found in 36.0% of patients, while problems with orientation, measured only by the MMSE, were found in 12.5% of patients. The three patients who were diagnosed with dementia after completing the study were shown to be far below the respective cut-off scores for cognitive impairment in both screening instruments (FTD-patient: ECAS total score: 65, MMSE total score: 22; bvFTD-patient: ECAS total score: 48, MMSE total score: 23; PPA-patient: ECAS total score: 28, MMSE total score: 17). One of the two patients with pending findings regarding dementia was below the respective cut-offs in both instruments (ECAS total score: 74, MMSE total score: 25), however the other patient only showed cognitive impairment using the ECAS (ECAS total score: 69, MMSE total score: 29).

**Fig 1:**
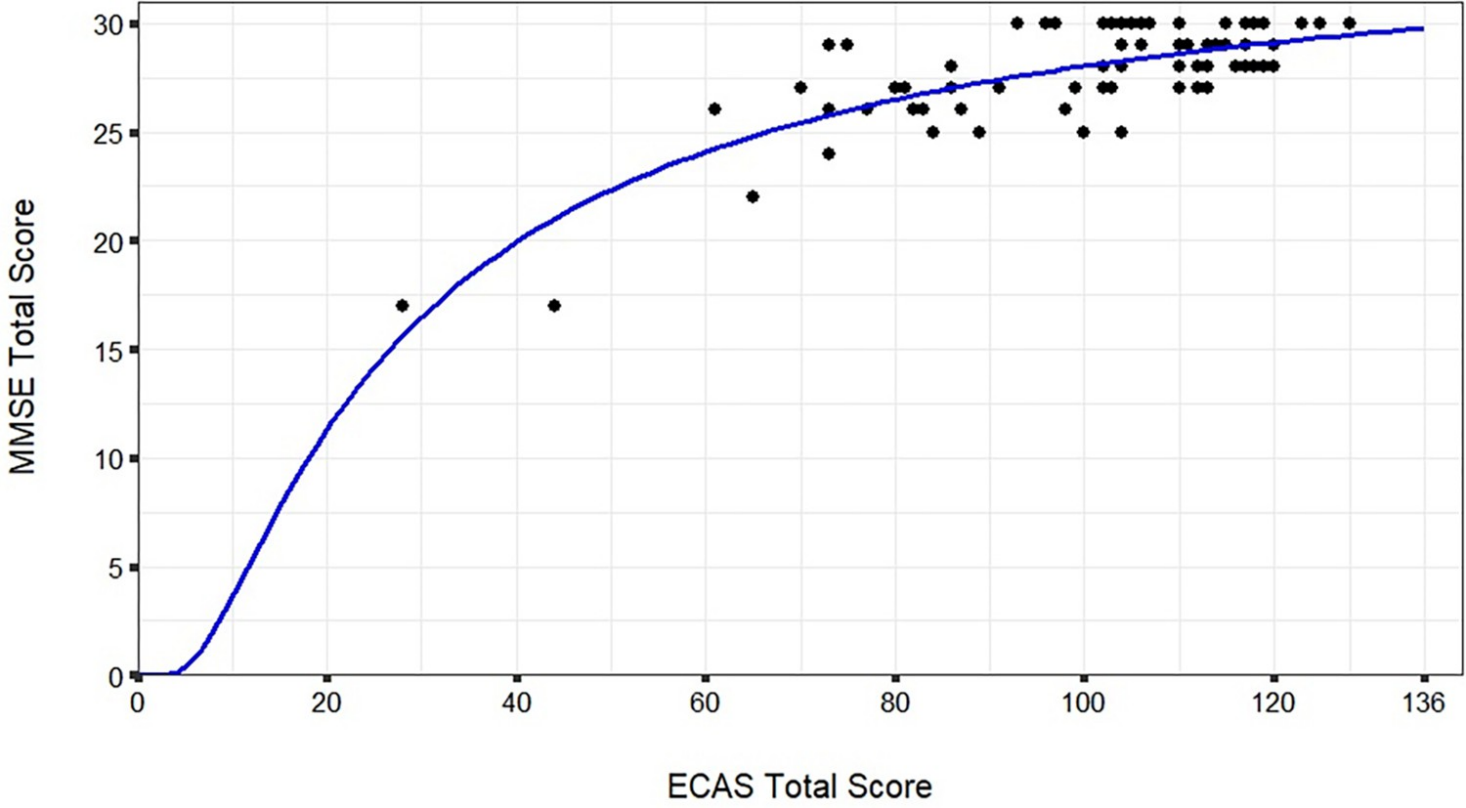
Prediction of MMSE score by the ECAS score. S-curve regression (F(1,73) = 156.41, *p* < .001, *R*^*2*^ = .68); ECAS = Edinburgh Cognitive and Behavioural ALS Screen. MMSE = Mini-Mental State Examination.

The relationship of the MMSE total score and the ECAS total score was shown to be non-linear (Fig 1). The MMSE total score was predicted by the ECAS total score (1/ECAS total score: β = -.83, *t* = -12.51, *p* < .001) by using an s-curve regression model (F (1,73) = 156.41, *p* < .001) with an adjusted R^2^ = .68 (plotted in Fig 1). The corresponding values of ECAS and MMSE calculated by this model are shown in Table 4, with the ECAS being more discriminative in the higher range of cognitive performance (range 80–136 ECAS total score), for which the MMSE does not define any cognitive impairment (corresponding to MMSE score 27–30).

**Table 4:** ECAS total scores and correspondent MMSE total scores according to s-curve regression model.

ECAS TOTAL SCORE	MMSE TOTAL SCORE
130–136	30
108–129	29
92–107	28
80–91	27
70–79	26
62–69	25
56–61	24
50–55	23
46–49	22
42–45	21
38–41	20
35–37	19
32–34	18
30–31	17
28–29	16
25–27	15
24	14
22–23	13
20–21	12
19	11
17–18	10
16	9
15	8
14	7
12–13	6
11	5
10	4
9	3
7–8	2
6	1
0–5	0

ECAS = Edinburgh Cognitive and Behavioural ALS Screen, MMSE = Mini-Mental State Examination. S-curve formula: y = exp(3.554634644851411–21.98030858426678 / x).

## Discussion

The purpose of this study was to compare the screening performance of the MMSE and the ECAS in ALS patients and to understand the relationship of both instruments. While the ECAS could be administered to the entire cohort, 89.3% of the patients were able to complete the MMSE in its original form. ALS patients who were not able to complete the MMSE had, as expected, a longer disease duration and were physically more impaired, however did not differ in general demographics, depressiveness, or cognition measured by the ECAS.

This study found a non-linear relationship between the performance on the ECAS and the MMSE of ALS patients with a relatively high R^2^ of .68. The cognitive domains of memory, language, executive functioning and visuospatial functioning, which all were included in both tests to a certain degree, showed relatively similar rates of impairment with 5 to 7.5% difference. Also, both MMSE and ECAS were able to identify all three patients with a diagnosed frontotemporal dementia variant with substantially decreased total scores, which could support the use of the MMSE in the detection or at least the monitoring of FTD in patients [16, 17]. If we assume a cut-off of 22 or 23 points [25, 26] for dementia in the MMSE, which fits our results, values of 46–55 in the total ECAS score may be indicative for dementia according to our calculated corresponding values. This, however, must be examined in a larger study regarding age and education.

While the rate of cognitively impaired patients was the same for both tests at approximately 23%, the agreement as to which patient is impaired and which is normal was relatively low. Only 53% of patients who showed cognitive impairment according to age- and education-based cut-offs of the ECAS in the total score were also impaired in the MMSE with an applicated cut-off of less than 27 points. Additionally, 47% of patients impaired in the ECAS total score were considered “normal” in the MMSE. An explanation could be the difference in tested cognitive domains in both tests. While orientation to time and place makes up a third of points in the MMSE, it is of no relevance in the ECAS. On the other hand, verbal fluency, a cognitive domain most typically found impaired not only in ALS patients [4–6, 27] but also in presymptomatic C9orf72 gene carriers [27, 28], and which was substantially decreased in 36% of patients in this study, cannot be examined by the MMSE. Also, the MMSE is known to suffer from ceiling effects [12, 25, 29, 30], especially in patients without full developed dementia [25, 29], which furthermore could explain the discrepancy in detection of cognitive impairment. In addition, meta-analyses show satisfactory values for sensitivity (77% [25] / 82% [26] / 85% [26]) and specificity (91% [25] / 90% [26] / 82% [26]) regarding the identification of dementia by the MMSE, but much lower values regarding the detection of mild cognitive impairment (sensitivity: 63%, specificity: 65%) [25]. Thus, application of the MMSE as a screening instrument for cognitive impairment in ALS patients is not ideal, and the usage of the ECAS should be preferred. If a shorter diagnostic tool must be used, the Montreal Cognitive Assessment (MoCA) was shown to be an acceptable alternative screening instrument for cognitive impairment in non-demented ALS patients, but also poses the problem of motor-dependence [30]. However, our results do not oppose the application of the MMSE as instrument for ruling out or monitoring dementia in ALS patients, which should be examined in further studies.

### Limitations

This cohort was tested in rather early stages of the disease with a still rather high motor capacity. Although the feasibility of the MMSE is expected to decline with further progression, present findings should not be extrapolated to late-stage patients. Although the rate of cognitive impairment and dementia matches the expected prevalence in ALS patients in general [1–3], the number of patients with cognitive impairments and dementia is low due to the overall relatively small number of patients. Further investigation of the relationship between the performance in MMSE and ECAS of ALS patients in a larger population, preferably with previously clearly defined subgroups with dementia, is recommended to potentially strengthen the current results.

## Conclusion

In summary, even though the MMSE is one of the most known and applied screening instruments, its application as a screener for cognitive impairment in early-stage ALS patients is not ideal, as it does not cover the full range of cognitive changes that may present in ALS patients and is also motor-dependent. The preferred tool should be the specially designed ECAS. However, if clinicians or researchers have no expertise in applying the ECAS or interpreting ECAS results, or if only the MMSE was used, it can be helpful to relate the ECAS score to the MMSE value or vice versa. The application of the MMSE as a screener to rule out dementia in ALS patients should be examined in further studies, also the creation of a cut-off for dementia in the ECAS should be considered, so that research studies and drug trials do not have to rely on screening instruments that are not suitable for ALS patients.
